# A Diamine-PEGylated Oleanolic Acid Derivative Induced Efficient Apoptosis through a Death Receptor and Mitochondrial Apoptotic Pathway in HepG2 Human Hepatoma Cells

**DOI:** 10.3390/biom10101375

**Published:** 2020-09-28

**Authors:** Fatin Jannus, Marta Medina-O’Donnell, Francisco Rivas, Luis Díaz-Ruiz, Eva E. Rufino-Palomares, José A. Lupiáñez, Andrés Parra, Fernando J. Reyes-Zurita

**Affiliations:** 1 Department of Biochemistry and Molecular Biology I, Faculty of Sciences, University of Granada, Av. Fuentenueva 1, 18071 Granada, Spain; fatin@correo.ugr.es (F.J.); luisdiazruiz96@gmail.com (L.D.-R.); evaevae@ugr.es (E.E.R.-P.); jlcara@ugr.es (J.A.L.); 2 Department of Organic Chemistry, Faculty of Sciences, University of Granada, Av. Fuentenueva 1, 18071 Granada, Spain; mmodonnell@ugr.es (M.M.-O.); aparra@ugr.es (A.P.)

**Keywords:** triterpenes, oleanolic acid, (PEG)ylated oleanolic acid, extrinsic apoptotic pathway, anti-tumor mechanisms, hepatocellular carcinoma

## Abstract

Hepatocellular carcinoma (HCC) is the most common type of liver cancer. Our recent studies have shown that the diamine-(PEG)ylated oleanolic acid (OADP) has strong anti-tumor effects in HCCs. In this study, we evaluated the anti-tumor mechanisms of OADP in the HepG2 HCC cell line. The cytotoxicity results showed that HepG2 cell viability was markedly reduced, with a very low 50% of cell growth inhibitory concentration (IC_50_, 0.14 µg/mL). We then investigated the anti-tumor mechanisms of OADP in HepG2 cells. The flow-cytometry analysis was used to evaluate cell apoptosis, indicating that 74–95% of cells were apoptotic. OADP caused cell cycle arrest in the G0/G1 phase and the loss of the mitochondrial membrane potential (MMP). Western blot analysis was performed to assess the expression levels of key proteins associated with the underlying molecular mechanism. The results showed the clear upregulation of caspase-8, caspase-9, caspase-3, Bak, p21, and p53, accompanied by the downregulation of Bcl-2. Similar results were obtained by the cotreatment with OADP and the c-Jun N-terminal kinase (JNK) inhibitor SP600125. Agents such as OADP, which are capable of activating extrinsic and intrinsic apoptotic pathways, may represent potential HCC cancer therapies.

## 1. Introduction

According to the World Health Organization (WHO), in 2018, cancer was the second-leading cause of death worldwide, responsible for 9.6 million deaths. The most common form of primary liver cancer is hepatocellular carcinoma (HCC), which represents approximately 75–85% of all liver cancer cases and is considered the second leading cause of cancer-associated death in East Asia and sub-Saharan Africa and the sixth most frequent cause of cancer-associated death in western countries [1].

HCC is generally only observed in patients with cirrhosis-damaged livers [2]. The primary risk factors for HCC include chronic infections, such as hepatitis C virus (HCV) and hepatitis B virus (HBV). Other risk factors include alcohol abuse, smoking, and obesity [3]. The primary therapeutic approaches for HCC patients include surgical resection, radiation therapy, liver transplantation, and chemotherapy [4]. However, several drugs have been developed in recent years to treat HCC, such as the tyrosine kinase inhibitors (TKIs) sorafenib, regorafenib, lenvatinib, and cabozantinib, which provide the efficient targeted therapy of HCC. Unfortunately, these drugs are also associated with adverse effects, including abdominal pain, hypertension, and hand-foot skin reactions [5].

Despite progress in the therapeutic approaches for HCC, the side effects caused by current treatment options necessitate the continued development of safe and efficient chemotherapy strategies. The use of natural products that are derived from plants represents a potential therapeutic approach to cancer treatment that could decrease the adverse effects of drugs. Therefore, many studies have focused on the identification of new anticancer drug treatments with natural origins [6]. Triterpenoids are the most abundant natural products, demonstrating anticancer and anti-tumor activities, and are often sources of anticancer agents, including oleanolic acid (OA).

Studies have shown that OA and its derivatives can exert anticancer activities in several cancer cell lines, including osteosarcoma, melanoma, breast cancer, and prostate cancer cells. OA also presents other pharmacological activities, such as anti-inflammatory, antidiabetic, hepatoprotective, and neuroprotective activities [7]. Our research group has applied polyethylene glycol (PEG)ylation techniques to OA, obtaining several OA derivatives [8,9,10]. PEGylation, which consists of the covalent bonding of a PEG polymer to an active biological agent, represents one of the most promising techniques for improving the therapeutic effects of drugs [11]. OADP is a diamine-PEGylated derivative of OA, obtained by performing a PEGylation reaction on the C-28 carboxyl group, using an amine-type PEG reagent (4,7,10-trioxatridecane-1,13-diamine, H_2_N-PEG-NH_2_) [8].

OA and its derivatives have been widely reported to induce apoptosis. OA and azaheterocyclic induce apoptosis through the alteration of Bax/Bcl2, resulting in the release of cytochrome-c and the activation of caspase-9 and caspase-3 in SMMC-7721 and BEL-7404 human HCC cells [12,13]. Another study showed that OA and ursolic acid induced apoptosis in four human liver cancer lines, including HepG2, Hep3B, Huh7, and HA22T, by enhancing caspase-8 and caspase-3 [14]. However, the effects of PEGylated oleanolic acid derivatives, such as OADP, on protein expression level in HCC cells and the underlying mechanisms of action have not yet been investigated. To study the anti-tumor effects of OADP on HCC cells, we used the HepG2 human carcinoma cell line because this cell line expresses the specific characteristics of hepatocytes, which are pathologically important to the progression of HCC.

Apoptosis, or programmed cell death, is defined as the active, physiological process of cell self-destruction. Two primary pathways have been described during apoptotic activation. The intrinsic pathway involves the release of pro-apoptotic factors, such as cytochrome-c, from the mitochondria, which activates the apoptotic mechanism through interaction with Apaf-1 and the stimulation of the initiator caspase-9. Caspase-9, in turn, proteolytically induces the activity of the executor caspase-3, one of the main proteases that participate in the apoptosis execution phase. In the extrinsic pathway, the activation of the death receptor stimulates the activation of the caspase-8 initiator, triggering downstream events by either directly activating caspase-3 or cleaving the Bid factor, which in turn, initiates the mitochondrial pathway. However, Bid activation has also been described as being mediated by c-Jun N-terminal kinase (JNK) [15,16]. Activated Bid targets the mitochondria, modulating other Bcl-2-like factors, such as Bax [17]. Furthermore, p53 has been identified as a key participant in the molecular mechanism associated with apoptosis induction, which involves both the transcriptional and non-transcriptional regulation of downstream effectors. For example, p53 induces apoptosis through the transcriptional upregulation of pro-apoptotic genes, such as Bax, and the transcriptional repression of the anti-apoptotic Bcl-2 [18]. p53 also translocates to the mitochondria prior to cytochrome-c release and pro-caspase-3 activation [19].

In our previous studies, our groupdescribed the anticancer properties of different triterpenoids, such as maslinic acid (MA) [20,21,22,23,24,25,26] and the OA derivative, 3-*O*-succinyl-28-*O*-benzyl oleanolate [27]. We also determined the underlying molecular mechanism associated with the anticancer effects of MA in Caco-2 and HT29 colon cancer cells. In Caco-2 colon cancer cells, MA treatment activated the extrinsic apoptotic pathway by inducing the cleavage of caspase-8 and caspase-3, increasing the levels of t-Bid, and decreasing the level of Bcl-2 [20,23]. Furthermore, we demonstrated that MA treatment induced apoptosis in human HT29 colon cancer cells by activating the intrinsic apoptotic pathway, induced by the expression of JNK, which also increased p53 levels and upregulated the expression of Bid and Bax, whereas Bcl-2 expression decreased. Finally, MA treatment also induced cytochrome-c release, due to mitochondrial disturbances, and increased the levels of cleaved caspase-9, -3, and -7 [23,24,25].

In this study, OADP was found to exhibit the marked inhibition of cell viability, inducing apoptosis and causing G0/G1 cell-cycle arrest, in a concentration- and time-dependent manner. We also investigated the in vitro effects of OADP on the HepG2 cell line and determined the underlying molecular mechanisms associated with OADP treatment. We determined that in HepG2 cells, OADP activated the proapoptotic response of the Bcl-2 protein family, such as Bak, and inhibited Bcl-2. OADP also activated transcription factors, such as p53 and p21. Finally, this compound induced the activation of initiator caspase-8 and caspase-9 and the effector caspase-3.

## 2. Materials and Methods

### 2.1. Materials

Dulbecco2019s modified Eagle medium (DMEM), foetal bovine serum (FBS), penicillin/streptomycin (Biowest, Nuaillé, France), dimethylsulfoxide (DMSO, Merck Life Science S.L., Madrid, Spain), and 3-(4,5-dimethylthiazol-2-yl)-2,5-diphenyltetrazolium bromide (MTT) were purchased from Thermo Fisher Scientific Inc. (Ward Hill, MA, USA). Caspase-3, Caspase-8, Caspase-9, Bcl-2, p53, Bak, and p21 antibodies were purchased from Santa Cruz Biotechnology (Santa Cruz, CA, USA). JNK inhibitor (SP600125) was purchased from Cell Signaling Technology, Inc. (Danvers, MA, USA). Caspase-8 inhibitor (IETD-CHO) and caspase-9 inhibitor (Ac-LEHD-CMK) were purchased from Merck Chemicals Ltd. (Padge Road, Beeston, UK). Secondary anti-rabbit, anti-mouse, and anti-goat antibodies and actin antibody were purchased from Santa Cruz Biotechnology (Santa Cruz, CA, USA). Culture flasks and well plates were obtained from VWR International, Ltd. (Radnor, PA, USA).

### 2.2. General Experimental Chemical Procedures

Measurements of nuclear magnetic resonance (NMR) spectra were made using VARIAN Inova unity (300 MHz ^1^H NMR), and VARIAN direct drive (400 and 500 MHz ^1^H NMR) spectrometers. The ^13^C chemical shifts were assigned with the aid of distortionless enhancement by polarization transfer (DEPT), using a flip angle of 135°. Infrared (IR) spectra were recorded on a MATTSON SATELLITE FTIR spectrometer. Optical rotations were measured with a Perkin-Elmer 241 polarimeter, at 25 °C. The purities of new compounds were determined by a WATERS ACQUITY UPLC system (ultra-performance liquid chromatography), coupled with a WATERS SYNAPT G2 HRMS spectrometer (high-resolution mass spectra) with electrospray ionization (ESI). The purities of all compounds were confirmed to be ≥95%. All reaction solvents and chromatography solvents were distilled prior to use. Commercially available reagents were used without further purification. Merck silica-gel 60 aluminium sheets (ref. 1.16835) were used for thin-layer chromatography (TLC), and spots were rendered visible by spraying with H_2_SO_4_–AcOH, followed by heating to 120 °C and visualizedvisualized under ultraviolet (UV) light at 254 nm. Merck silica-gel 60 (0.040–0.063 mm, ref. 1.09385) was used for flash chromatography. CH_2_Cl_2_ (Fisher, ref. D/1852/17) or n-hexane (Merck, ref. 1.04374), with increasing amounts of acetone (Fisher, ref. A/0600/17) or EtOAc (Fisher, ref. E/0900/17), were used as eluents (all solvents were of analytical reagent grade purity). The PEG reagent 4,7,10-trioxatridecane-1,13-diamine (H_2_N-PEG-NH_2_, CAS Number 4246-51-9) was purchased from Sigma–Aldrich. Boc_2_O (CAS Number 24424-99-5) was also purchased from Sigma–Aldrich.

The plant material, a specimen of the plant of *Olea europaea* L. (order Lamiales, family Oleaceae), was collected in Almegíjar, Granada, Spain, in May 2001. Laura Baena, from the herbarium of the University of Granada, identified this plant. A voucher specimen (53489-1-1) was deposited at the University of Granada Herbarium, Granada, Spain.

### 2.3. Isolation of OA

OA was isolated from solid olive oil production wastes, which were extracted successively in a Soxhlet, with hexane and EtOAc. OA was purified from hexane extracts, using column chromatography over silica gel and eluting with CH_2_Cl_2_/acetone mixtures of increasing polarity [28].

### 2.4. PEGylation Reaction of OA

A solution of di-tert-butyl dicarbonate (Boc_2_O, 2.75 mmol) in dried CH_2_Cl_2_ (2 mL) was added slowly, dropwise, to a solution of 4,7,10-trioxatridecane-1,13-diamine (H_2_N-PEG-NH_2_, 6.8 mmol) in CH_2_Cl_2_ (20 mL). The reaction mixture was maintained at room temperature (rt) for 12 h, and then diluted with water and extracted three times with CH_2_Cl_2_. The organic layer was dried with anhydrous Na_2_SO_4_, and the solvent was removed under reduced pressure, producing the diamine-Boc-PEGylated derivative (H_2_N-PEG-NH-Boc, 85%), blocked in an amino group [8].

In a flask (20 mL), the reagent H_2_N-PEG-NH-Boc (0.45 mmol) was dissolved in dimethylformamide (5 mL) and afterwards, OA (2 mmol), 1-hydroxy-7-azabenzotriazole (HOAt, 3 mmol), (7-Azabenzotriazol-1-yloxy)tripyrrolidinophosphonium hexafluorophosphate (PyAOP, 2 mmol), and *N*,*N*-diisopropylethylamine (DIPEA, 8 mmol), were added. The reaction mixture was heated to 100 °C for 12 h, diluted with water, and extracted three times with CH_2_Cl_2_. The organic layer was dried with anhydrous Na_2_SO_4_, and the solvent was removed under reduced pressure. Finally, the residue was purified by column chromatography, using n-hexane/ethyl acetate as the solvents, to yield the OA-diamine-Boc-PEGylated derivative (94%) [8].

**Figure 1:**
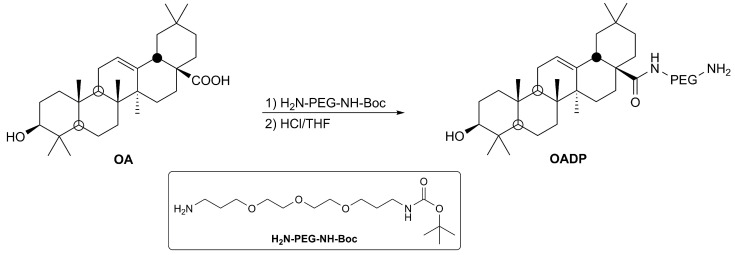
Semi-synthesis of a diamine-PEGylated derivative of oleanolic acid (OADP).

This OA-diamine-Boc-PEGylated derivative (0.3 mmol) was dissolved in tetrahydrofuran (THF, 20 mL), and then concentrated HCl (37%, 2 mL) was added. The reaction mixture was maintained at rt for 24 h, then diluted with water and extracted three times with CH_2_Cl_2_. The organic layer was dried with anhydrous Na_2_SO_4_, and the solvent was removed under reduced pressure. Finally, the residue was purified by column chromatography, using n-hexane/ethyl acetate as solvents, and to yield OADP (95%) (Figure 1) [8].

### 2.5. Drugs

OADP was dissolved, at 5 mg/mL, in DMSO. A stock solution was stored at −20 °C. Before the experiments, this solution was diluted in the cell culture medium, to the desired concentration. Cytometric analyses were performed at the OADP the 20%, 50%, and 80% cell growth inhibitory concentrations (IC_20_, IC_50_ ± and IC_80_, respectively) for 24, 48, and 72 h. Western blot analyses were performed after 72 h of treatment at the OADP IC_50_ and IC_80_ values.

### 2.6. Cell Culture

The HepG2 human HCC cell line (ECACC cell line no. 85011430) and the WRL68 non-tumor human embryo liver cells (EACC no. 89121403) were supplied by the cell bank of the University of Granada, Granada, Spain. Cell lines were cultured in DMEM, supplemented with 2 mM glutamine, 10,000 units/mL penicillin, and 10 mg/mL streptomycin, containing 10% decomplemented FBS, at 37 °C, in an atmosphere of 5% CO_2_ and 95% of humidity. Cells were grown in the absence of OADP, for 24 h before treatment. In all experiments, we used sub-confluent monolayer cells.

### 2.7. Cytotoxicity Assay

OADP treatment effects on the proliferation of HepG2 cells were assessed using the mitochondrial membrane potential (MTT) assay. Cell viability was determined by measuring the absorbance of the MTT dye in living cells. MTT cleaves the tetrazolium ring to produce formazan, which absorbs at 570 nm.

For this assay, 1.5 × 10^3^ HepG2 cells and 8.0 × 10^3^ WRL68 cells, were grown in each well of a 96-well plate and incubated with increasing concentrations (0–20 µg/mL) of OADP, for 24 and 48 h for HepG2 cells, and 72 h for HepG2 and WRL68 cells. After incubation, the media was removed, and 100 µL of MTT solution (0.5 mg/mL), in a mixture of 50% phosphate-buffered saline (PBS) and 50% medium, was added to each well. After 2 h of incubation, formazan was dissolved in 100 µL DMSO. Relative cell viability, with respect to the untreated control cells, was measured according to absorbance at 570 nm, using a plate reader (Tecan Sunrise MR20-301, TECAN, Salzburg, Austria). OADP showed high levels of cytotoxicity at the very lowest tested concentrations. Therefore, we examined the mechanisms through which this cytotoxicity occurred. Therefore, apoptosis, cell cycle, and mitochondrial membrane potential (MMP) analyses were performed, and we also investigated the activated molecular mechanisms associated with cell death in HepG2 cells.

### 2.8. Flow Cytometric Assay

#### 2.8.1. Apoptosis Analysis

Apoptosis was quantified by flow cytometry, using a FACScan (fluorescence-activated cell sorter) flow-cytometer (Coulter Corporation, Hialeah, FL, USA), analyzing between 5000 and 10,000 events. HepG2 cells were plated at 1.5 × 10^5^ cells per well on a 24-well plate, in 2 mL of medium, and treated with OADP for 24, 48, and 72 h, at the IC_20_, IC_50_, and IC_80_ values. Cells were collected and resuspended in binding buffer (10 mM HEPES/NaOH, pH 7.4, 140 mM NaCl, 2.5 mM CaCl_2_). Annexin V-fluorescein isothiocyanate (FITC) conjugate (1 µg/mL) was then added and incubated for 30 min at rt in the dark. Just before the FACS analysis, we stained the cells with 20 µL of 1 mg/mL propidium iodide (PI) solution. All experiments were performed two times, in triplicate, including the control.

#### 2.8.2. Cell Cycle Analysis

To determine the main alterations in the cell cycle profiles, particularly DNA ploidy, we used FACS. We measured those alterations at 488 nm on an Epics XL flow cytometer (Coulter Corporation, Hialeah, FL, USA), analyzing between 2500 and 10,000 events. Cell subpopulations with differing DNA contents (cells in G0/G1, S or G2/M phase) were visualized. In addition, we were able to distinguish the population size and the nucleus fractions in each phase of the cell cycle, and calculated the DNA ratios for each identified nuclear subpopulation. According to the previous protocol, the percentages of cells in different phases of the cell cycle were detected by PI staining. HepG2 cells, seeded at a density of 1.5 × 10^5^ cells/well, were plated into 24-well plates with 2 mL of medium. For 24, 48 and 72 h, the cells were treated with OADP at the IC_20_, IC_50_ and IC_80_ concentrations, previously obtained during the cell proliferation activity test. Cells were washed twice with cold PBS, trypsinised, centrifuged at 1500 rpm for 5 min, and then washed twice with cold PBS. After the second centrifugation, the cells were resuspended in 1 × TBS staining buffer (10 mM Tris, 150 mM NaCl), followed by the addition of Vindelov buffer (100 mM Tris, 100 mM NaCl, 10 mg/mL RNase, 1 mg/mL propidium iodide (PI), pH 8). Just before the measurements, the total DNA content was stained with 1 mg/mL of PI. We used Multicycle software to determine the percentages of cells in different phases of the cell cycle (G0/G1, S, and G2/M). All experiments were performed two times, in triplicate, including the control.

#### 2.8.3. Mitochondrial Membrane Potential Analysis

We studied mitochondrial damage using a FACScan flow cytometer. We used dihydrorhodamine (DHR), which becomes oxidizedz to the highly fluorescent rhodamine (Rh123), to measure the MMP. Fluorescence spectroscopy, using excitation and emission wavelengths of 500 and 536 nm, respectively, was used to monitor rhodamine formation. Intracellular measurements of MMP were determined by the cytometry of Rh123. HepG2 cells were plated at 1.5 × 10^5^ cells per well in 24-well plates, in 1.5 mL of medium, and treated with the OADP for 24, 48, and 72 h at the previously calculated IC_20_, IC_50_, and IC_80_ concentrations. After treatment, the medium was removed, and fresh medium containing DHR was added, at a final concentration of 5 µg/mL. The medium was removed, and the cells were washed and resuspended in PBS containing 5 µg/mL of PI, after 30 min incubation. Finally, the fluorescence intensities of Rh123 and PI were determined using a FACScan flow cytometer (Coulter Corporation, Hialeah, FL, USA), analyzing between 4000 and 10,000 events. All experiments were performed two times, in triplicate, including the control.

### 2.9. Western Blotting Analysis

HepG2 cells (1.5 × 10^4^) were treated with OADP at the previously calculated IC_50_ and IC_80_ concentrations for 72 h. Cells were also co-incubated for 72 h with the IC_50_ concentrations of OADP, a caspase-8 inhibitor (IETD-CHO, 18 nM), a caspase-9 inhibitor (Ac-LEHD-CMK, 70 nM), and JNK inhibitor (SP600125, 25 µM). After the treatments, cells were washed twice with PBS and resuspended in lysis buffer (20 mM Tris/acetate, pH 7.5, 1 mM EDTA, 1 mM EGTA, 1% Triton X-100, 1 mM orthovanadate, 270 mM sucrose, 1 mM sodium glycerophosphate, 5 mM sodium fluoride, 1 mM sodium pyrophosphate, 5 mM β-mercaptoethanol, 1 mM benzamidine, 35 µg/mL PMSF, and 5 µg/mL leupeptin). Samples were homogenized, ultrasonicated, and incubated on ice for 20 min before centrifuging at 12,000× *g* for 15 min. Supernatants were assayed to determine the protein concentration. The protein concentration was determined by the Bradford method. For Western blot analyses, a 25–50 µg sample of total proteins was used. Proteins were separated on 15% sodium dodecyl sulfate (SDS)-polyacrylamide gel and transferred to a polyvinylidene difluoride membrane. The membranes were blocked by incubation in TBS buffer containing 0.1% Tween and 5% milk powder, for 1 h at rt, and washed with TBS buffer containing 0.1% Tween. Membranes were blotted overnight, at 4 °C, with primary antibodies (Mouse monoclonal anti-caspase-8 (1/200 dilution), goat polyclonal anti-caspase-3 (1/600 dilution), mouse monoclonal anti-Bcl-2 (1/200 dilution), rabbit polyclonal anti-caspase9 (1/500 dilution), rabbit polyclonal anti-p53 (1/4000 dilution), rabbit polyclonal anti-Bak (1/800 dilution), and rabbit polyclonal anti-p21 (1/500 dilution)). The blots were then washed 3 times with TBS-0.1% Tween and developed with peroxidase-linked secondary antibodies for 1 h at rt, with the following dilutions (1/3000, 1/3000, 1/3000, 1/13,000, 1/13,000, 1/13,000, 1/12,000). All antibodies were obtained from Santa Cruz Biotechnology (Santa Cruz, Inc., CA, USA). Blots were then washed 3 times with TBS-0.1% Tween and once with TBS. Consequently, all blots were revealed using the ChemiDoc XRS Image System (Bio-Rad Laboratories, Hercules, CA, USA). Finally, the quantification of protein bands was performed using Multi-Gauge program (Fuji Film Europe, TK Tiburg, Holland).

### 2.10. Hoechst-Stained Fluorescence Microscopy

Morphological changes were analyzed by Hoechst-stained fluorescent microscopy. Therefore, 15 × 10^4^ HepG2 cells were plated on coverslip in 24-well plates. After 24 h, OADP was added and cells were incubated for 72 h at their respective IC_50_ and IC_80_ concentrations. The cells were then washed twice with PBS, treated in cold MeOH for 3 min, washed in PBS, and incubated in 500 µL Hoechst solution (50 ng/mL) in PBS for 15 min in the dark. The samples were visualized by fluorescent microscopy (DMRB, Leica Microsystems, Wetzlar, Germany) with a DAPI filter.

### 2.11. Statistical Analysis

Data are represented as the mean ± standard deviation (SD). For each experiment, the Student’s *t*-test 2019 was used for statistical comparisons against untreated control cells. A limit of *p* ≤ 0.05 was used to determine significant differences. Key: *p* < 0.05 (*), *p* < 0.01 (**) and *p* < 0.001 (***). All data shown here are representative of at least two independent experiments, performed in triplicate.

## 3. Results

### 3.1. Effects of a Diamine-PEGylated Derivative of Oleanolic Acid (OADP) on HepG2 Proliferation

To evaluate the cytotoxic effects of OADP (Figure 1) on the HepG2 cell line, we incubated these cells at increasing concentrations (0–20 µg/mL) of OADP for 24, 48, and 72 h. Cell viability was analyzed by MTT assay, based on formazan dye concentrations.

**Figure 2:**
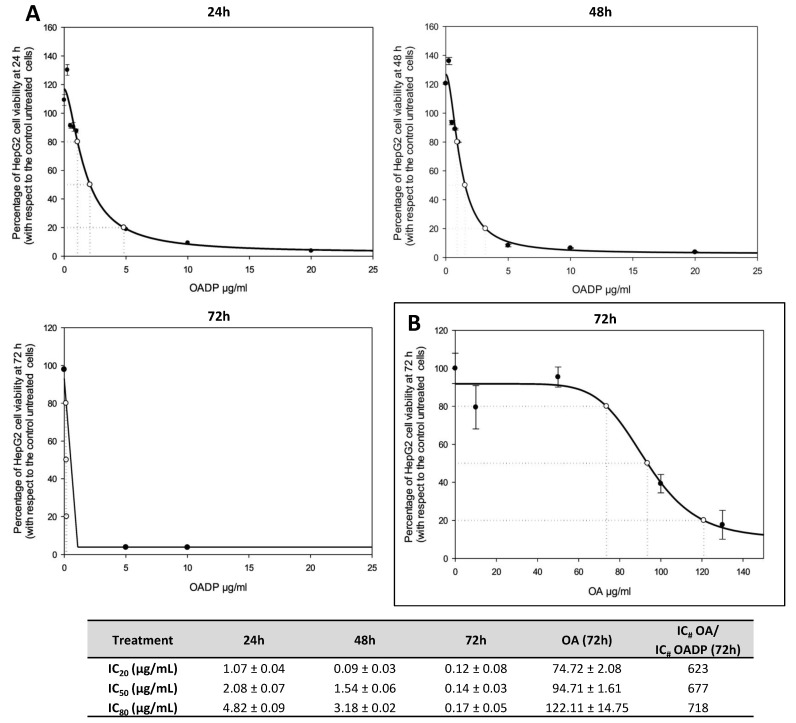
(**A**) Effects of OADP on the viability of the HepG2 human hepatocarcinoma cells. OADP treatment was applied for 24, 48, and 72 h, in the range of 0 to 20 µg/mL, and each point represents the mean ± S.D. of at least two independent experiments, performed in triplicate. (**B**) Effects of OA on the viability of HepG2 cells, after treatment for 72 h. IC_20_, IC_50_, and IC_80_ represent the concentrations required for 20%, 50%, and 80% growth inhibition, respectively. OA, oleanolic acid; OADP, diamine-PEGylated OA.

The percentage of growth inhibition in the presence of various concentrations of OADP for HepG2 cells was determined as the percentage of viable treated cells relative to viable, untreated control cells. As shown in Figure 2, OADP induced significant cell death, in a concentration- and time-dependent manner. The OADP concentrations required for 20% growth inhibition (IC_20_), 50% growth inhibition (IC_50_), and 80% growth inhibition (IC_80_) were determined for all incubation times. The IC_20_ and IC_50_ concentrations were less than 3 µg/mL for all three analyzed times. The IC_80_ concentration was less than 5 µg/mL for all three times. The IC_50_ concentrations obtained for the 24 and 48 h time points were 45–62 times lower than those for OA. These concentrations were especially low after 72 h of OADP treatment, with values lower than 1 µg/mL (IC_20_ = 0.12 ± 0.01 µg/mL; IC_50_ = 0.14 ± 0.03 µg/mL; IC_80_ = 0.17 ± 0.05 µg/mL). The IC_50_ concentration for this compound (OADP), at 72 h, was 677 times lower than its precursor (OA), and 718 times lower at the IC_80_ concentration. This cytotoxicity appears to be selective for the HepG2 hepatic tumor cell line. Thus, in the WRL68 non-tumor liver cells of human embryos, the IC_50_ value at 72 h of incubation with OADP was 5.47 ± 0.12 µg/mL, which implies a cytotoxicity 39 times lower in this line than in the HepG2 tumor cell line.

The extremely low concentrations obtained in these tests led us to determine whether cytotoxicity was due to the activation of apoptosis and whether any cytostatic effects were associated with the cytotoxic response. Therefore, the following cytometric assays were performed, including the determination of apoptosis through annexin-V-FITC staining, cell cycle analysis using PI staining, and the evaluation of changes in MMP. These concentrations and times were selected for the rest of the assays: cell cycle analysis, characterization of apoptosis, and changes in mitochondrial membrane potential. IC_50_ and IC_80_ concentrations obtained for the 72 h treatment time point, were selected for the analysis of protein expression level by Western blot analysis because these concentrations resulted in the strongest effects.

### 3.2. Characterisation of the Apoptotic Effects of OADP by Flow Cytometry

The main objective of developing new drugs capable of blocking cell proliferation and inducing apoptosis is to obtain anticancer agents that can inhibit the growth of tumor cells and arrest the cell cycle. The loss of cytoplasmic membrane asymmetry occurs early during apoptosis, caused by the translocation of phosphatidylserine (PS) from the leaflet of the internal membrane to the external membrane, exposing PS to the external environment, where it can be recognized by macrophage cells [29]. The exposed PS is recognized by annexin V phospholipid-binding protein, which binds to and fluorescently labels apoptotic cells.

**Figure 3:**
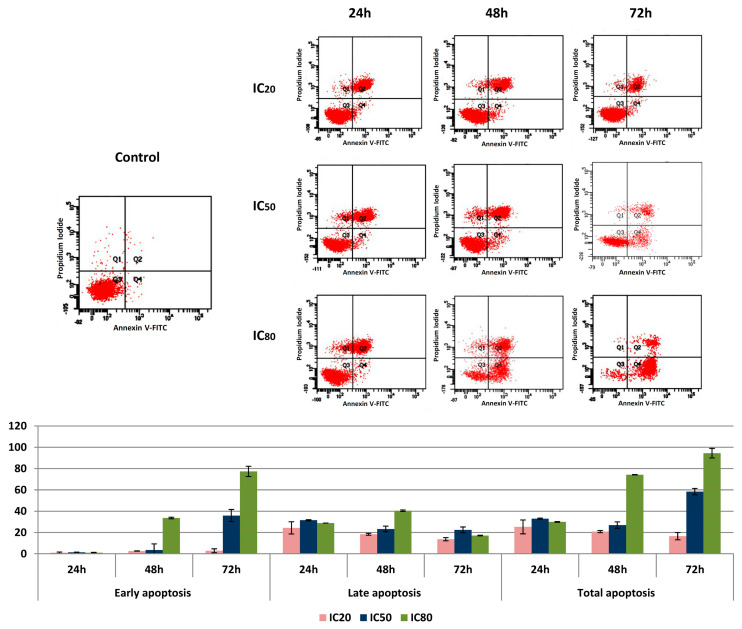
Flow cytometric analysis of Annexin V-FITC staining and propidium iodide (PI) accumulation after the exposure of HepG2 cells to OADP for 24, 48, and 72 h. The cell line was treated at concentrations equal to the IC_20_, IC_50_, and IC_80_ values. Top: Diagrams of annexin V/PI flow cytometry. The right quadrants of each diagram represent apoptotic cells (Q2, late apoptosis; Q4, early apoptosis). Bottom: Flow cytometry analysis of Annexin V-FITC staining and PI accumulation. Values represent the mean ± S.E.M of duplicate independent experiments, performed in triplicate.

We performed apoptosis cytometric studies in the HepG2 cell line, treated with OADP at the IC_20_, IC_50_, and IC_80_ concentrations, for 24, 48, and 72 h. Apoptotic tests were performed by double staining with Annexin V, and conjugated to FITC and PI. Apoptosis percentages were determined for Annexin V-FITC/PI stained cells using FACS analysis (Figure 3). This double-staining method differentiated four cell populations: normal cells (Annexin V^−^ and PI^−^), early apoptotic cells (Annexin V^+^ and PI^−^), late apoptotic cells (Annexin V^+^ and PI^+^), and necrotic cells (Annexin V^−^ and PI^+^).

Treatment of HepG2 cells with OADP was shown to induce apoptosis, in a time- and concentration-dependent manner. The lowest percentage of total apoptosis was observed at the IC_20_ concentration, with similar values for all three time points, (close to 25%, consisting of 1% early apoptosis and 24% late apoptosis). At the IC_50_ concentration, the total percentage of apoptosis obtained was close to 30% (3% early apoptosis plus 27% late apoptosis), at the 24 and 48 h time points, whereas 60% of apoptosis was observed after 72 h of treatment. The highest percentage of apoptosis was obtained at the IC_80_ concentration, after 48 h, which was 74% (34% early apoptosis plus 40% late apoptosis), and 72 h, which was 94% (77% early apoptosis plus 17% late apoptosis). In addition, the percentages of the necrotic populations were not remarkable.

The low IC_20_, IC_50_, and IC_80_ values, and the good percentages of apoptosis observed indicated that OADP could represent a promising anticancer drug; therefore, we decided to explore the apoptotic trigger mechanisms. The effects of this product on the cell cycle were studied, below. The distribution of cells in different cell cycle phases was analyzed after 24, 48, and 72 h of incubation at the mentioned concentrations, based on the incorporation of PI.

### 3.3. OADP Induces Cell Cycle Arrest in the G0/G1 Phase

In response to treatment with the triterpene derivative OADP, cell proliferation is suppressed through cytotoxic and cytostatic effects. To determine the potential cytostatic effects associated with the cytotoxic response, we analyzed the cell cycle distribution and cell cycle arrest. Flow cytometry was used to sort PI-stained cells and to measure DNA ploidy and alterations in cell cycle profiles in HepG2 cells treated with the IC_20_, IC_50_, and IC_80_ OADP values for 24, 48 and 72 h. The DNAs content are directly proportional to PI fluorescence, which allows the determination of the percentage of cells in each cell cycle phase. This method allows the cell subpopulations presenting different DNA contents to be visualized. Changes in DNA concentrations are characteristic of apoptosis and cell cycle arrest.

**Figure 4:**
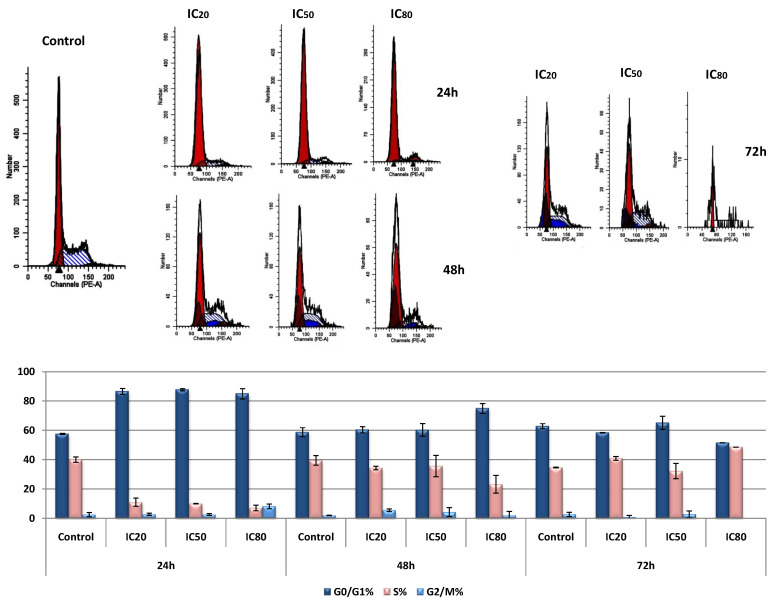
Changes occurred in the percentages of cells in each of the cell cycle phases, compared with untreated control cells. HepG2 cells were treated with OADP at the IC_20_, IC_50_, and IC_80_ concentrations. Cell cycle analysis was performed after PI staining. Cells in the G0/G1 phase (dark blue bar), S phase (pink bar), and G2/M phase (light blue bar) were counted. Values represent the mean ± SEM of at least two independent experiments, performed in duplicate.

Alterations in the HepG2 cell cycle, induced by OADP treatment, are shown in Figure 4. After 24 h of incubation, the important growth arrest at the G0/G1 phase was observed (57.5% in the control, 86.5% at IC_20_, 87.6% at IC_50_, and 84.9% at IC_80_). These percentages represented a 47% increase in the proportions of cells in this phase of the cycle compared with untreated control cells. These increased proportions of cells in the G0/G1 phase were accompanied by a concomitant decrease in the proportions of cells in S phase. The proportions of cells in S phase were markedly reduced, from 40% in control cells to 10.9%, 9.9%, and 7.0% following OADP treatments at IC_20_, IC_50_, and IC_80_ concentrations, respectively.

However, treatment at 48 h and 72 h, using the same concentrations, produced a slight arrest in the growth of the G0/G1 cell cycle. After 48 h of treatment, only a 15% increase in cells was observed in this cell cycle at the IC_80_ concentration, whereas after 72 h of incubation, only a 5% increase was found in this phase at the IC_50_ concentration. However, a 15% increase in the cell population was detected in the S phase at the IC_80_ concentration.

These changes in the percentages of cells arrested at different stages suggested that the G0/G1 phase may be associated with an OADP-induced cytostatic process at the beginning of the treatment process, which disappears after 48 h of treatment. Future trials will be necessary to verify this possibility. The arrest in S phase, which was observed after 72 h of treatment, may be a consequence of apoptosis induction.

### 3.4. OADP Treatment Causes Changes in the Mitochondrial Membrane Potential

The two primary apoptotic pathways are the intrinsic mitochondrial apoptotic pathway and the extrinsic apoptotic pathway. The first pathway causes mitochondrial disruption and involves the loss of MMP. In contrast, the second pathway induces apoptosis without initial MMP changes. To determine the apoptosis mechanism involved in the apoptotic response of HepG2 cells, we analyzed MMP using Rh123 staining. This compound is a membrane-permeable, fluorescent cationic dye that is selectively taken up by the mitochondria and its fluorescence is proportional to MMP.

**Figure 5:**
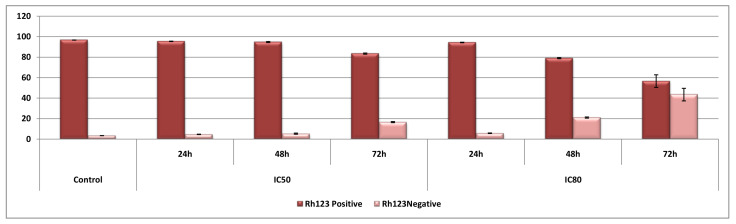
Percentage of HepG2 Rh123-positive cells, relative to untreated control cells (control), after treatment with OADP for 24, 48, and 72 h, at the IC_50_ and IC_80_ concentrations. Values are expressed as the mean ± SD of two independent experiments, performed in triplicate.

To determine the possible mechanisms involved in the apoptotic response of HepG2 cells induced by OADP, we analyzed the MMP of these cells after incubation with OADP, at IC_20_, IC_50_, and IC_80_ concentrations, for 24, 48, and 72 h of incubation time (Figure 5).

The results obtained showed that 24 h after OADP treatment, no changes in MMP were observed. However, after 48 h of incubation at the IC_80_ concentration, the percentage of Rh123-negative cells increased by 18% compared with the untreated control cells. No changes were detected for the other tested concentrations. After 72 h of incubation at the IC_50_ concentration, the percentage of Rh123-negative cells increased by 15% compared with untreated controls cells, whereas this percentage increased by 40% at the IC_80_ concentration.

These results clearly showed that no changes in MMP occurred after the initial treatments with OADP. Subsequent changes in MMP occurred gradually, with the largest change observed after 48 h of treatment at the IC_50_ concentration, and visible changes at the IC_80_ concentration after 72 h of treatment. These results could be related to the initial activation of the extrinsic apoptotic pathway, followed by the subsequent, secondary activation after 72 h of incubation of the intrinsic apoptotic pathway in response to the initial extrinsic apoptotic signals, which likely intensifies the initial apoptotic signal produced by OADP treatment.

### 3.5. OADP Triggers the Activation of Caspases-8, -3 and -9 to Induce Apoptosis

Cancer occurs when the cells prevent the occurrence of apoptosis and begin to proliferate uncontrollably. However, during the discovery and development of anticancer drugs, new molecules have been identified that are capable of activating the cellular molecular mechanism of apoptosis induction. Apoptosis is regulated by a family of cysteine proteases named caspases.

The activation of the caspase cascade is one of the most important processes that occur during the induction of apoptotic cell death. Our cytometric results showed a significant increase in apoptotic cell death in the HepG2 cell line after 72 h of OADP treatment. Therefore, we examined the level of the initiator caspases, caspase-8 and caspase-9, and the executor caspase, caspase-3, at the 72-h time point. To determine whether OADP induced apoptosis in HepG2 cells using the extrinsic apoptotic pathway (caspase-8) or the intrinsic apoptotic pathway (caspase-9), HepG2 cells were treated at the IC_50_ and IC_80_ concentrations of OADP for 72 h. We also analyzed the level of the caspase-3 effector, which increases in response to the activation of the intrinsic and extrinsic apoptotic pathways.

To verify that the apoptotic pathway was activated and to determine the apoptotic implications of caspase-8, caspase-9, and JNK activation, we additionally used the caspase-8 inhibitor (IETD-CHO), caspase-9 inhibitor (Ac-LEHD-CMK), and JNK inhibitor (SP600125), to block their activated molecular pathways. HepG2 cells were co-incubated with the caspase-8 inhibitor (18 nM), the caspase-9 inhibitor (70 nM), and JNK inhibitor (25 µM), and the corresponding IC_50_ OADP concentration, for 72 h. The protein expression levels of the apoptotic markers caspase-8, caspase-9, and caspase-3 were individually examined using western blot analysis.

Our results showed evidence that OADP significantly activates caspase-8 expression levels (1.2-fold increase at IC_50_, 2.0-fold increase at IC_80_, and 2.2-fold increase at IC_50_ with the concomitant inhibition of JNK). However, no significant changes at the IC_50_ OADP concentration were detected in conjunction with the caspase-9 or caspase-8 inhibitors. Increases in caspase-8 levels were accompanied by the cleavage of pro-caspase-8, under all conditions and concentrations tested.

**Figure 6:**
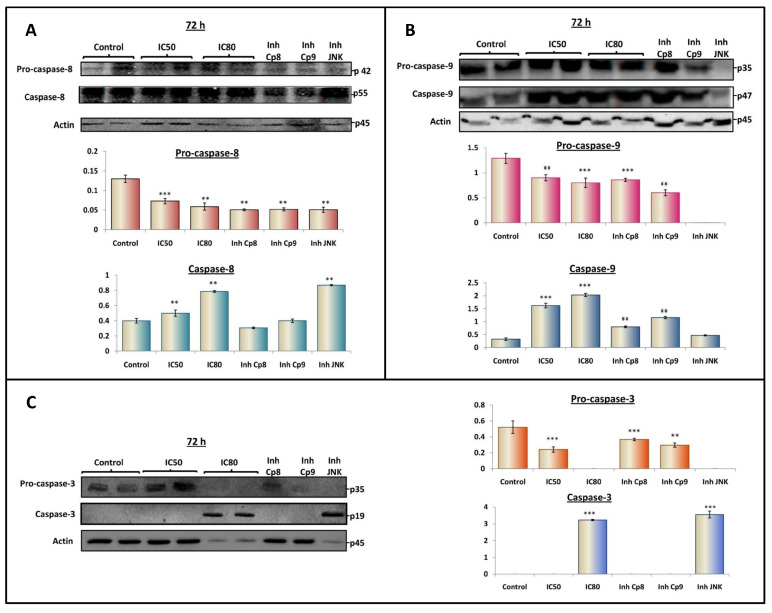
Western blotting analysis of the levels of pro-caspase-8, caspase-8 (**A**); pro-caspase-9, caspase-9 (**B**); and pro-caspase-3, caspase-3 (**C**). HepG2 cells were treated with OADP at IC_50_ and IC_80_ concentrations. ‘Inh Cp8’, ‘Inh Cp9’ and ‘Inh JNK’, correspond with the caspase-8 inhibitor, caspase-9 inhibitor, and JNK inhibitor, respectively. The levels of protein expression are expressed as the arbitrary intensity units for each band relative to the arbitrary intensity units of actin. The values represent the mean ± SD of at least three separate experiments. Key: (**) *p* < 0.01 and (***) *p* < 0.001, with respect to the untreated control cells.

These results indicated that OADP-induced apoptosis occurs initially through the activation of the extrinsic apoptotic pathway (level of active caspase-8), followed by the secondary activation of the intrinsic apoptotic pathway. JNK inhibition increased the level of caspase-8, likely because the inhibition of this part of the pathway increases the apoptotic signal through caspase-8 activation. (Figure 6A).

The level of active caspase-9 protein increased in HepG2 cells exposed to OADP (by 3.0-fold at IC_50_ and by approximately 3.4-fold at IC_80_). Although the expression level of active caspase-9 decreased significantly in the presence of caspase-8 and caspase-9 inhibitors, in the presence of the JNK inhibitor, pro-caspase 9 was not detected and a lower level of active caspase-9 was detected, although these changes were not significant compared with the untreated control. These increases in the active caspase-9 were accompanied by the consequent decrease in pro-caspase-9 levels, under the conditions and concentrations analyzed (Figure 6B).

In this case, our results indicated that the JNK inhibition produced the lowest levels of pro-caspase-9 and active caspase-9. JNK inhibition likely completely inhibits the secondary activation of the intrinsic apoptotic pathway, which is mediated by caspase-9.

Finally, the expression level of active caspase-3 was remarkably improved in HepG2 cells in response to OADP combined with JNK inhibitor treatment (3.2-fold at the IC_80_ concentration and 3.5-fold at the IC_50_ concentration combined with the JNK inhibitor). However, no detectable levels of active caspase-3 were observed at the IC_50_ concentration, either with or without caspase-8 or caspase-9 inhibitors. The undetectable levels of active caspase-3 under these conditions may be due to the reduced proportion of apoptotic cells compared with those obtained at the IC_80_ concentration. These increases in caspase-3 levels were accompanied by the marked cleavage of pro-caspase-3. The decrease in pro-caspase-3 levels was visible under all concentrations and conditions assayed, although pro-caspase-3 was not detectable at the IC_80_ concentration, nor in the treatment at the IC_50_ concentration combined with the JNK inhibitor, likely because almost all pro-caspase-3 was activated under these conditions and concentrations (Figure 6C).

### 3.6. OADP Triggers the Activation of p53 and p21^Cip1/Waf1^ to Induce Apoptosis

The role of the p53 tumor-suppressor protein is well-known, regulating various phenomena, including cell growth, cell cycle arrest, and apoptosis induction. In contrast, JNK phosphorylates and regulates the activity of transcription factors, such as p53 [30,31].

Several mechanisms, both transcriptional and non-transcriptional, have been identified during the regulation of apoptosis induction by p53. For example, p53 induces apoptosis through the transcriptional upregulation of pro-apoptotic genes, such as Bax or Bak, and the transcriptional repression of the anti-apoptotic Bcl-2 [18]. Furthermore, p53 controls the gene expression of other regulators proteins, such as p21^Cip1/Waf1^, which are involved in regulating the advance and arrest of the cell cycle [32]. This effect could be related to the G0/G1 cell-cycle arrest produced in response to OADP treatment.

**Figure 7:**
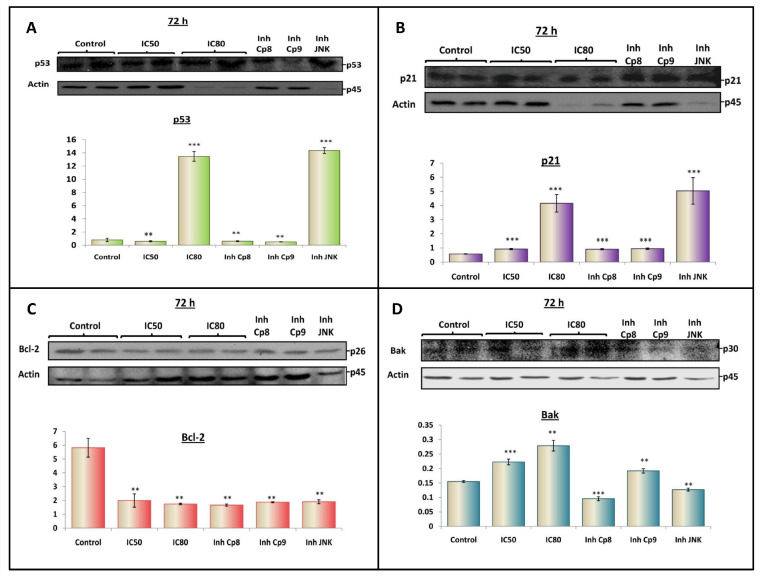
Western blot analysis of the protein levels of p53 (**A**), p21 (**B**), Bcl-2 (**C**), and Bak (**D**). HepG2 cells were treated with OADP at IC_50_ and IC_80_ concentrations. ‘Inh Cp8’, ‘Inh Cp9’ and ‘Inh JNK’ correspond to the combined treatment with caspase-8 inhibitor, the caspase-9 inhibitor, and the JNK inhibitor, respectively. Protein expression levels are expressed as units of arbitrary intensity for each band, compared to the units of arbitrary intensity for the actin band. Values represent the mean ± SD of at least three separate experiments. Key: (**) *p* < 0.01 and (***) *p* < 0.001, with respect to the untreated control cells.

We studied the effects of OADP on p53 expression level at the IC_50_ and IC_80_ concentrations, alone, and at the IC_50_ concentration in combination with inhibitors of caspase-8, caspase-9, and JNK, to better understand the molecular mechanisms that underlie the activation of apoptosis in HepG2 cells, in response to OADP treatment. The results showed the clear induction of p53 level at the IC_80_ concentration and during the co-treatment with the JNK inhibitor, at the IC_50_ concentration. p53 induction resulted in protein expression levels 17–18 times that observed in the untreated control cells (Figure 7A). However, at the IC_50_ concentration and in combination with the inhibition of caspase-8 or caspase-9, no significant changes were observed. These results are in agreement with the results that were previously found during the analysis of the cell cycle and apoptosis, in which the initial treatment resulted in stronger cell cycle arrest in the G0/G1 phase than was observed after 72 h of incubation. This result is likely due to the initial differentiation process induced by the activation of p53/p21 in response to OADP treatment, which was inhibited after long incubation times, activating the apoptosis process. However, p53 is also an important apoptosis inducer, which is reflected by the increased percentage of apoptosis induction observed for the IC_80_ concentration compared with the IC_50_ concentration. Finally, JNK inhibition resulted in the activation of p53 at the IC_50_ concentration, likely due to the disappearance of p53 inhibition, which is mediated by JNK activation.

p21^Cip1/Waf1^ is a 21-kDa protein that acts as a cyclin-dependent kinase inhibitor and triggered in response to p53 protein activation. p53 activation tends to be described in response to stress stimuli, inhibiting the cyclin-cdk2 complex kinase activity required for the transition from the G1 to S phases of the cell cycle. At the initial time point, this protein is activated by p53, which blocks cell cycle progression, inducing cell arrest, cell differentiation, and DNA repair [33]. If the signal or stress stimuli continue over time, the apoptotic signal overlaps with p21 function, and the cell eventually enters apoptosis.

To determine the effects of OADP treatment, we evaluated the expression level of p21 in HepG2 cells, at the concentrations and conditions mentioned above. The results showed that the p21 protein levels increased 1.6-fold at the IC_50_ concentration and 7.2-fold at the IC_80_ concentration, compared with the level in untreated control cells. Similar results were obtained between IC_50_ concentrations and IC_50_ concentrations combined with caspase-8 and caspase-9 inhibitors. However, when combined with JNK inhibition, the level of p21 increased by 9-fold compared with the level in control cells (Figure 7B). The activation of p21 is fully correlated with p53 activation, which is most likely due to the dependence of p21 levels on the transcriptional activity of p53. The activation of p21, in response to OADP treatment, could explain the cell cycle arrest observed in the presence of OADP, and the possible activation of differentiation processes. Future analysis will be necessary to verify this finding.

### 3.7. OADP Triggers the Activation of Bcl-2 and Bak to Induce Apoptosis

To gain a complete picture of the activated molecular mechanism associated with apoptosis induction by OADP, we determined the effects of this compound on the expression levels of the Bcl-2 protein family. The expression levels of the pro-apoptotic protein Bak and the anti-apoptotic protein Bcl-2 were analyzed after 72 h of incubation with OADP at the IC_50_ and IC_80_ concentrations, alone, and at the IC_50_ concentration in combination with the caspase-8, caspase-9, and JNK inhibitors.

The secondary activation of the intrinsic apoptotic pathway, to strengthen the primary extrinsic apoptotic signal, has been extensively described in the apoptotic response to triterpenes compounds [34,35]. Caspase-8 and JNK can activate Bid protein (pro-apoptotic protein in the Bcl-2 protein family). Bid active, or t-Bid, targets the mitochondria to modulate other Bcl-2-like factors, such as Bcl-2 or Bak, which ultimately results in the loss of MMP and mitochondrial membrane permeability, releasing mitochondrial factors, such as cytochrome-c or Apaf-1 proteins, which activate caspase-9 through apoptosome formation.

Our results revealed a decrease in Bcl-2 protein levels, in a concentration-dependent manner, at IC_50_ and IC_80_ concentrations (OADP decreased Bcl-2 protein levels by 66% at IC_50_, and by 70% at IC_80_). Similar results were obtained at the IC_50_ concentration combined with caspase-8, caspase-9 or JNK inhibitors. These results may be associated with the inhibition of Bcl-2 protein expression, independent of JNK activity, likely due to p53 and/or caspase-8 activity (Figure 7C).

Bak protein levels increased in a concentration-dependent manner (1.44-fold at IC_50_ and approximately 2-fold at IC_80_) following OADP treatment. In contrast, Bak protein levels decreased by 38% at the IC_50_ concentration combined with caspase-8 inhibitor, a slight decrease was observed at the IC_50_ concentration combined with the caspase-9 inhibitor, and decreased by 18% at the IC_50_ concentration combined with the JNK inhibitor. These results may be explained by the dependence of Bak activation on caspase-8 or JNK activities, whereas the inhibition of caspase-9 resulted in a lower decrease in this protein level, likely because caspase-9 is upregulated by this protein. Therefore, the inhibition of caspase-9 had a minor effect on its expression (Figure 7D).

### 3.8. OADP Induces Apoptotic Morphological Changes (Hoechst-Stained)

**Figure 8:**
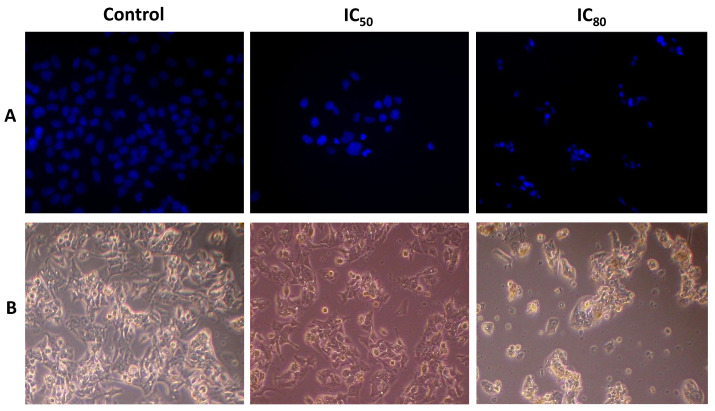
Morphological changes in HepG2 cells after exposure to OADP for 72h, at IC_50_ and IC_80_ concentrations. Cells were examined using fluorescence microscopy with a DAPI filter, following Hoechst-stained as described in the Section 2 (**A**). Phase-contrast light microscopy cell images (**B**).

The Hoechst procedure stains nuclei containing nicked DNA, a characteristic that cells exhibit in apoptotic cell death. Morphological analysis of Hoechst-stained cells, in HepG2 cells, indicated that they had undergone remarkable morphological changes (Figure 8). At the IC_50_ concentration of OADP, cells showed typical apoptotic changes, including cell shrinkage, chromatin condensation, and loss of normal nuclear architecture. At the IC_80_ concentration, the disruption of the cell-membrane integrity was more prominent. Microscopic observation of fluorescence after Hoechst staining showed that a significant number of cells treated with OADP acquired apoptotic features, as evident by nuclear fragmentation.

## 4. Discussion

The lack or failure of apoptosis activation mechanisms and programmed cell death disrupts cell homeostasis, which is a primary cause of cancer induction. Therefore, many efforts and studies have focused on the identification of compounds capable of reactivating apoptosis mechanisms, to utilize this cell death process in the clinical treatment of cancer [36,37]. Our results showed that the triterpene derivative (OADP) is a promising compound that is capable of inducing apoptosis in a concentration- and time-dependent manner, at very low concentrations (Figure 3), producing the significant inhibition of cell viability. Compared with its natural precursor (OA), OADP is 718 times more effective in HepG2 cells, after 72 h of treatment [8]. Furthermore, OADP induces morphological changes that are characteristic of apoptosis [8,23,25,26,27,38], such as chromatin condensation and fragmentation, as evidenced by fluorescence microscopy. This effect appears to be selective for the tumor cell line, because the cytotoxicity found in the WRL68 non-tumor human embryonic liver cells was 39 times lower than in HepG2 cells.

Furthermore, the antiproliferative activity of OADP appears to be associated with its cytotoxicity, together with the activation of a cytostatic effect, as evidenced by the induction of cell-cycle arrest in the G0/G1 phase (Figure 4). We also discovered that during the initial treatment stage (24 and 48 h), no changes in the MMP were observed, which agrees with the activation of the extrinsic apoptotic mechanism. However, after 72 h of treatment, we observed the significant loss of MMP, which is likely associated with the secondary activation of the intrinsic apoptotic mechanism, to increase the initial apoptotic signal (Figure 5). Therefore, these results suggested that OADP activates the extrinsic apoptotic pathway first, followed by the intrinsic apoptotic pathway.

Because of the activation of the apoptosis mechanism, a family of cysteine proteases, known as caspases, was activated [39]. We can distinguish two groups or types of caspases, initiator caspases and effector caspases. Initiator caspases, such as caspase-8 and -9, are activated in response to apoptotic stimulation and cause the downstream activation of effector caspases, such as caspase-3 (main effector caspase), caspase-6, or caspase-7, which induce typical apoptotic changes, such as chromatin condensation and fragmentation, cell shrinkage, and loss of cell membrane asymmetry [40].

Two major apoptotic pathways are triggered during apoptotic cell death: the extrinsic apoptotic pathway, which is induced by external signals, and the intrinsic apoptotic pathway, which is induced by cellular stress stimuli [41]. To determine which apoptotic pathway was activated in response to OADP in HepG2 cells, we evaluated the expression level of key proteins involved in the apoptosis mechanisms of the extrinsic and intrinsic apoptotic pathways. First, we analyzed the expression level of the initiating caspases-8 and -9 and found that after 72 h of incubation with OADP, the expression levels of these caspases increased. However, no significant changes were observed in the expression level of caspase-8 at the IC_50_ OADP concentration combined with the caspase inhibitors. Levels similar to those observed for the IC_80_ OADP concentration were achieved by combining the IC_50_ OADP concentration with the JNK inhibitor, likely because JNK may be involved in the potential secondary activation mechanism of the intrinsic apoptotic pathway. When JNK was inhibited, the apoptotic signal associated with caspase-8 activation increased (Figure 6B). Caspase-9 levels increased in response to IC_50_ and IC_80_ OADP concentrations in a similar manner as caspase-8 levels. Although no significant changes were observed at the IC_50_ OADP concentration combined with caspase-8 or -9 inhibitors, the reduction of pro-caspase-9 and caspase-9 levels was observed when the IC_50_ OADP concentration was combined with the JNK inhibitor. Finally, caspase-3 activation was observed at the IC_80_ OADP concentration and the IC_50_ OADP concentration combined with the JNK inhibitor, likely because these conditions promoted the activation of apoptosis pathways compared with the IC_50_ OADP concentration, either alone or in combination with the caspases-8 and -9 inhibitors.

The co-treatment with inhibitors of caspase-8, -9, and JNK, combined with the IC_50_ OADP concentration revealed that caspase-8 level was independent of these other factors, whereas caspase-9 increased when JNK was inhibited, indicating that caspase-9 level depends on caspase-8 and JNK expression level. Therefore, we suggest that the cleavage and activation of caspase-9 activation were induced by caspase-8 and/or JNK. Based on these results, we suggest that the apoptotic mechanism activated in response to OADP involved the extrinsic apoptotic activation route, first, followed by the secondary intrinsic activation pathway, to enhance the initial apoptotic signal, as described in the apoptotic response to other triterpene compounds, such as MA and the oleanolic acid derivative CDDO [7].

In our previous study, we described the activation of the intrinsic and extrinsic apoptotic pathway following MA treatment in the HT-29 and Caco-2 cell lines. The activation of the extrinsic and intrinsic apoptotic pathways in response to many OA derivatives has been reported. 3-*O*-acetyloleanolic acid induced the increase of caspase-8 and caspase-3 levels in human colon carcinoma HCT-116 cells. Similarly, HIMOXOL (methyl 3-hydroxyimino-11-oxoolean-12-en-28-oate) also induced the activation of poly-ADP-ribose polymerase (PARP)-1 in MDA-MB-231 breast cancer cells [42,43]. Meanwhile, the OA derivative SZC015 induced a mitochondrial apoptotic pathway in human breast cancer cells by activating cleaved caspase-3, caspase-9, cytosolic Cyt C, and PARP and increasing the Bax/Bcl-2 ratio [44]. Additional studies demonstrate that OA methyl ester induced the intrinsic apoptotic pathway in PC-3 prostate cancer cells [45]. However, Koetjapic acid, a natural triterpenoid, induced apoptosis in HCT 116 colorectal carcinoma cells by activating both the extrinsic and intrinsic caspases, which increased the hypoxia-inducible factor (HIF)-1α, mitogen-activated protein (MAP)/extracellular signal-related kinase (ERK)/JNK, and Myc/Max signaling pathways and decreased the nuclear factor (NF)-κB signaling pathway [34].

This secondary activation of the intrinsic apoptotic route can be initiated by caspase-8 activation. Thus, caspase-8 targets the BH3-only protein Bid for cleavage, generating the activated t-Bid fragment, which activates pro-apoptotic Bcl-2 proteins, such as Bak or Bad, and ultimately results in the loss of the mitochondrial external membrane permeability and MMP and induces the activation of caspase-9 and caspase-3. Furthermore, Bid activation has been described as dependent on JNK and mediated by p53 [46], both of which may be involved in the proposed apoptosis mechanism.

JNK also activates molecular pathways during cellular proliferation or death [47], activating the downstream control of proteins related to cell cycle and apoptosis, such as p53, p21 or cyclin D. The main target of JNK (c-Jun) inhibits p-53-mediated cell cycle arrest, promoting p53-mediated apoptosis. c-Jun has also been reported to function as a direct repressor of p53-mediated gene transcription [48].

The involvement of p53 and p21^Cip1/Waf1^ has been reported to be important for apoptosis induction in many hepatoma cell lines triggered by triterpenoids, such as saikosaponin [49] or OA [50]. Several mechanisms have been identified for the induction of apoptosis by p53, involving both the transcriptional and/or non-transcriptional regulation of downstream effectors. In this study, we evaluated p53 expression levels in response to OADP after 72 h of incubation, which resulted in the significant increase of p53 level at the IC_80_ concentration and the IC_50_ concentration combined with the JNK inhibitor (Figure 7A). This effect may be due to JNK activation, which can suppress the transcription of the p53 gene [48]. When JNK expression is inhibited, p53 is transcribed, significantly increasing its cellular levels. Furthermore, the induction of p53 expression may explain the increasing levels of p21 because p21 is transcribed by p53, and the results obtained for these protein levels (Figure 7B) were very similar to those obtained for p53 protein levels. The expression of p53 and p21 proteins may be closely related to the significant cell cycle arrest observed during the initial treatment stage (24 h) with OADP. However, this cell cycle arrest effect disappears, due to the continued apoptotic stimulation, after 48 and 72 h of treatment. A similar mechanism has been described for other triterpenoids, such as Saikosaponin-d (SSd), which inhibited cell proliferation in the HepG2 and Hep3B HCC cell lines and DU145 prostate cancer cells, through the upregulation of p53 and p21, cell cycle arrest in the G0/G1 phase, and apoptosis induction [32,49].

JNK and p53 interact with the Bcl-2 family of anti-apoptotic and pro-apoptotic proteins, regulating the apoptotic process. JNK can act directly on the Bcl-2 family of proteins to induce the activation of the mitochondrial pathway. JNK phosphorylates members of the Bcl-2 family of proteins, such as Bcl-2, which inactivates their anti-apoptotic functions, and can induce proapoptotic Bcl-2 proteins containing only the BH3 domain, such as Bid or Bim, which can mediate the activation of apoptosis by Bax or Bak [51]. In contrast, several p53 mechanisms of apoptosis induction have been identified that involve both the transcriptional and/or non-transcriptional regulation of downstream effectors. p53 induces the upregulation of Bax expression and the transcriptional repression of Bcl-2 [18]. In addition, the physical inhibition by p53 of the anti-apoptotic proteins Bcl-2 and Bcl-xL was described, and its participation in the formation of oligomers with Bax/Bak proteins results in the permeabilisation of the external mitochondrial membrane, causing the release of cytochrome-c into the cytosol [52]. Treatments with the JNK inhibitor have been reported to strongly induce CD95 expression, inducing apoptosis in six different tumor cell lines through p53- and p21-mediated G2/M cell cycle arrest, and caspase-8 and caspase-3 cleavage [53].

Next, we evaluated the expression levels of the Bcl-2 and Bak proteins, in response to treatment with OADP, to determine their participation in the molecular apoptosis mechanisms induced by this compound. Our results, after 72 h of treatment with OADP, showed the downregulation of the anti-apoptotic protein Bcl-2, accompanied by the upregulation of the pro-apoptotic protein Bak (Figure 7C,D). Co-treatment with the IC_50_ OADP concentration and caspase-8, caspase-9, and JNK inhibitors, resulted in no changes in Bcl-2 levels, which remained lower in all conditions compared with untreated control cells, which may be caused by Bcl-2 levels being regulated by both JNK and p53. The inhibition of caspase-8 and JNK results in decreased Bak, whereas the inhibition of caspase-9 produced a smaller effect, so that Bak expression level depends on the activities of caspase-8 and JNK, and not on caspase-9, likely because caspase-9 is downregulated by Bak.

In summary, our results showed the clear activation of the initiating caspases-8 and -9 and the effector caspase-3, the increase in cell cycle and apoptosis regulating proteins p53 and p21, the upregulation of the proapoptotic protein Bak, and the downregulation of Bcl-2 anti-apoptotic protein. In the presence of the caspase-8 inhibitor, we found the downregulation of caspase-8, caspase-9, caspase-3, p53, p21, and Bak, indicating that the expression levels of these proteins were directly associated with caspase-8 activation. Therefore, we were able to demonstrate that apoptosis activation in HepG2 cells, in response to OADP treatment, occurs through the activation of the extrinsic apoptotic mechanism, mediated by caspase-8. In the presence of the caspase-9 inhibitor, we found the downregulation of caspase-9, caspase-8, caspase-3, p53, and p21, and the reduced inhibition in Bak, indicating that the intrinsic apoptosis mechanism was also activated, likely to enhance the initial extrinsic apoptotic signal. Finally, with JNK inhibition, we identified large increases in the levels of caspase-8, caspase-3, p53, and p21 proteins, whereas we observed decreased Bak and caspase-9 levels, indicating that JNK was likely involved in regulating the expression levels of these proteins, and is the likely link between the two apoptotic activation routes. The activation of the JNK-mediated intrinsic apoptotic pathway, in response to caspase-8 activation, has been previously described [54]. Bcl-2 level protein decreased in all treatment conditions assayed. These results are consistent with the different apoptosis mechanisms described in the literature [34,35].

## 5. Conclusions

The results reported here demonstrated that OADP triggered apoptosis and cell cycle arrest in the HepG2 cancer cell line, in a concentration- and time-dependent manner. These results showed that OADP is a powerful anticancer compound, capable of causing cell cycle arrest during the early stages of incubation, and the activation of the two main apoptotic pathways, the extrinsic and intrinsic apoptotic routes, in the HepG2 cell line, inducing the inhibition of cancer cell proliferation at very low concentrations (Figure 2). Finally, we observed that OADP treatment results in the loss of MMP after 72 h of treatment, indicating that the activation of the intrinsic apoptotic pathway is secondary to the activation of the extrinsic apoptotic pathway.

**Figure 9:**
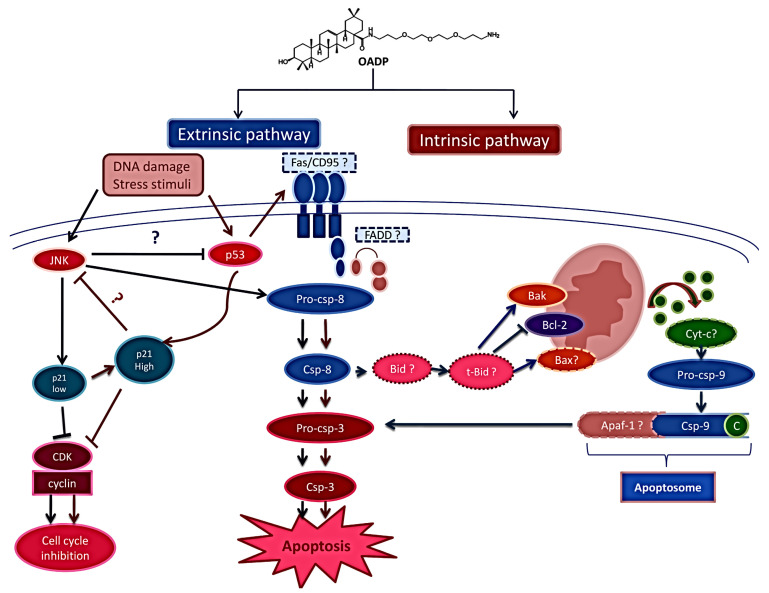
Mechanism of action underlying apoptosis induced by OADP in HepG2 cells after 72 h of treatment, at IC_50_ and IC_80_ concentrations, alone, and IC_50_ concentrations combined with the JNK inhibitor (SP600125). Dark blue arrows represent IC_50_ apoptotic pathway, and dark red arrows represent apoptotic pathways associated with the IC_80_ concentration and the IC_50_ concentration in combination with the JNK inhibitor (SP600125).

Based on these results, we propose the following mechanism for the apoptotic effect of OADP on HepG2 cells (Figure 9). First, OADP induces JNK and p53 protein level increase, causing cell cycle arrest and activating p21 during the early stages. Furthermore, we can assume that OADP initially triggers the extrinsic apoptotic pathway, initiated by caspase-8, which activates the caspase-3 executor. The activation of caspase-8 and JNK causes the secondary activation of the intrinsic apoptotic pathway, through the upregulation of Bak and the downregulation of Bcl-2, triggering the mitochondrial apoptotic response, which subsequently activates caspase-9. Both the initial activation of caspase-8 and the secondary activation of caspase-9 lead to the cleavage of caspase-3. Finally, the complete process induces apoptosis in HepG2 cells, which is significant after 48 and 72 h of OADP treatment, as revealed by the FACS analysis. The apoptotic results of OADP suggest that this compound could serve as an effective compound during the treatment of HCC.

To our knowledge, this is the first time that the ability of OADP to induce efficient apoptosis through the extrinsic and intrinsic apoptotic pathways has been highlighted in the HepG2 human cell line, which is a particular type of HCC. Interestingly, our results indicated that OADP can act as an effective anticancer agent against the HepG2 human cell line, in vitro. The effects of OADP must be studied in vivo and in other HCC cell lines.
