# Correction: Jannus et al. A Diamine-PEGylated Oleanolic Acid Derivative Induced Efficient Apoptosis through a Death Receptor and Mitochondrial Apoptotic Pathway in HepG2 Human Hepatoma Cells. *Biomolecules* 2020, *10*, 1375

**DOI:** 10.3390/biom16050691

**Published:** 2026-05-07

**Authors:** Fatin Jannus, Marta Medina-O’Donnell, Francisco Rivas, Luis Díaz-Ruiz, Eva E. Rufino-Palomares, José A. Lupiáñez, Andrés Parra, Fernando J. Reyes-Zurita

**Affiliations:** 1 Department of Biochemistry and Molecular Biology I, Faculty of Sciences, University of Granada, Av. Fuentenueva 1, 18071 Granada, Spain; fatin@correo.ugr.es (F.J.); luisdiazruiz96@gmail.com (L.D.-R.); evaevae@ugr.es (E.E.R.-P.); jlcara@ugr.es (J.A.L.); 2 Department of Organic Chemistry, Faculty of Sciences, University of Granada, Av. Fuentenueva 1, 18071 Granada, Spain; mmodonnell@ugr.es (M.M.-O.); aparra@ugr.es (A.P.)

In the original publication [1], there was a mistake in Figure 3 as published. During the final stage of figure preparation (resolution enhancement), the IC_50_ panel corresponding to 48 h was inadvertently duplicated for 72 h, leading to an overlap between the 48 h and 72 h IC_50_ subpanels. The figure has now been corrected in the updated manuscript provided with this correction request. The authors state that the scientific conclusions are unaffected. This correction was approved by the Academic Editor. The original publication has also been updated.

**Figure 3:**
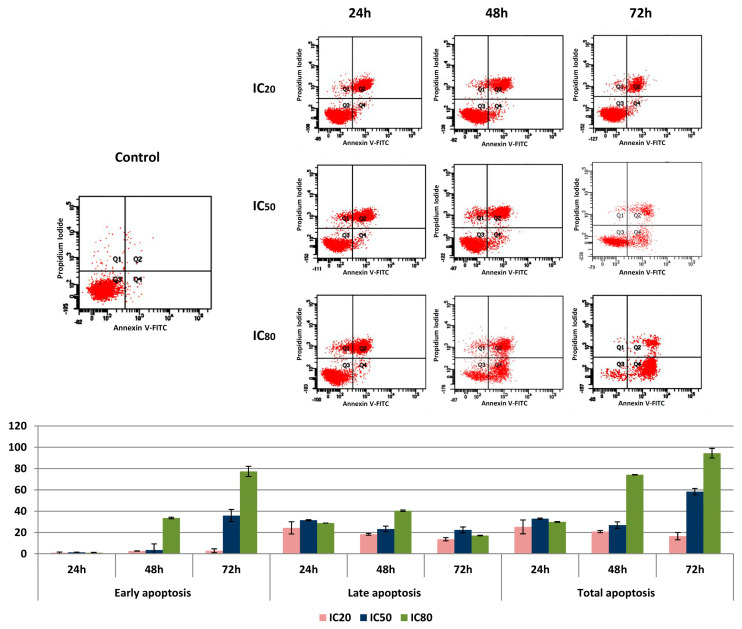
Flow cytometric analysis of Annexin V-FITC staining and propidium iodide (PI) accumulation after the exposure of HepG2 cells to OADP for 24, 48, and 72 h. The cell line was treated at concentrations equal to the IC_20_, IC_50_, and IC_80_ values. Top: Diagrams of annexin V/PI flow cytometry. The right quadrants of each diagram represent apoptotic cells (Q2, late apoptosis; Q4, early apoptosis). Bottom: Flow cytometry analysis of Annexin V-FITC staining and PI accumulation. Values represent the mean ± S.E.M of duplicate independent experiments, performed in triplicate.
